# A Strain Decoupling Packaging Strategy for High‐Fidelity Ultrathin Silicon Shape Sensors for Soft Medical Robotics

**DOI:** 10.1002/advs.202518733

**Published:** 2026-02-26

**Authors:** Hao Liu, Masahito Takakuwa, Michitaka Yamamoto, Yiwen Wang, Tomoyuki Yokota, Takao Someya, Toshihiro Itoh, Seiichi Takamatsu

**Affiliations:** ^1^ Department of Precision Engineering Graduate School of Engineering The University of Tokyo Tokyo Japan; ^2^ Institute of Engineering Innovation Graduate School of Engineering The University of Tokyo Tokyo Japan; ^3^ Department of Electrical and Electronic Engineering and Information Systems Graduate School of Engineering The University of Tokyo Tokyo Japan; ^4^ School of Systems Science and Industrial Engineering Thomas J. Watson College of Engineering and Applied Science State University of New York at Binghamton Binghamton New York USA

**Keywords:** shape sensing, soft robotics, strain decoupling packaging, ultrathin silicon

## Abstract

Reliable soft sensors are essential for precise control, real‐time feedback, and safe operation in soft medical robots. Ultrathin silicon piezoresistive sensors offer flexibility, durability, and high sensitivity, but protecting them from external stress while maintaining softness remains challenging. Existing packaging strategies often compromise flexibility and induce excessive strain, leading to device failure. Here, we introduce an oil‐lubricated slidable packaging approach that enables high‐fidelity shape sensing of ultrathin silicon gauges by mechanically isolating the sensing element within a dual‐layer film. This design decouples the sensor from the packaging, minimizing bending strain on the sensing element. Experimental results show that the sensor maintains stable and reliable signals under large applied strains, with fast response (<0.40 s), low hysteresis (∼3 %), and durability over more than 10 000 bending cycles at a 2 mm radius. The sensor was successfully integrated into a flexible endoscope, enabling precise shape detection under continuous 360° unidirectional bending, demonstrating feasibility for complex 3D perception. This work addresses long‐standing packaging challenges in ultrathin flexible devices and provides a structurally protective strategy for reliable shape sensing in flexible electronic systems.

## Graphical abstract

Reliable soft sensors are essential for precise control in soft medical robots such as endoscopes. We developed ultrathin silicon piezoresistive sensors and their oil‐lubricated slidable packaging, which enables high‐fidelity shape sensing of silicon gauges by mechanically isolating the sensing element within a dual‐layer film. This design decouples the sensor from the packaging, improving the mechanical reliability of the sensor.

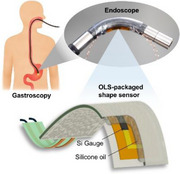

## 1 Introduction

Soft shape sensors have recently gained significant attention for controlling soft actuators in medical robotics, including endoscopic and other minimally invasive surgical devices, because they offer improved safety and human compatibility compared with rigid sensing systems [1, 2, 3]. These flexible sensors and actuators are often encapsulated in elastomeric materials or films and integrated onto the robot surface as soft robotic skin [4, 5, 6]. In contrast, conventional robotic systems rely on electric motors and rigid encoders to monitor rotational motion. In soft medical robotic systems such as endoscopes, shape sensors, and their packaging must provide highly accurate deformation detection and remain reliable under large external stresses and repeated mechanical loading, often exceeding tens of thousands of cycles, because these devices operate inside the human body [7]. However, achieving the required softness while ensuring human safety, protecting fragile soft sensors, and maintaining sensing accuracy, mechanical compliance, and long‐term stability remains a significant challenge [7, 8].

**FIGURE 1:**
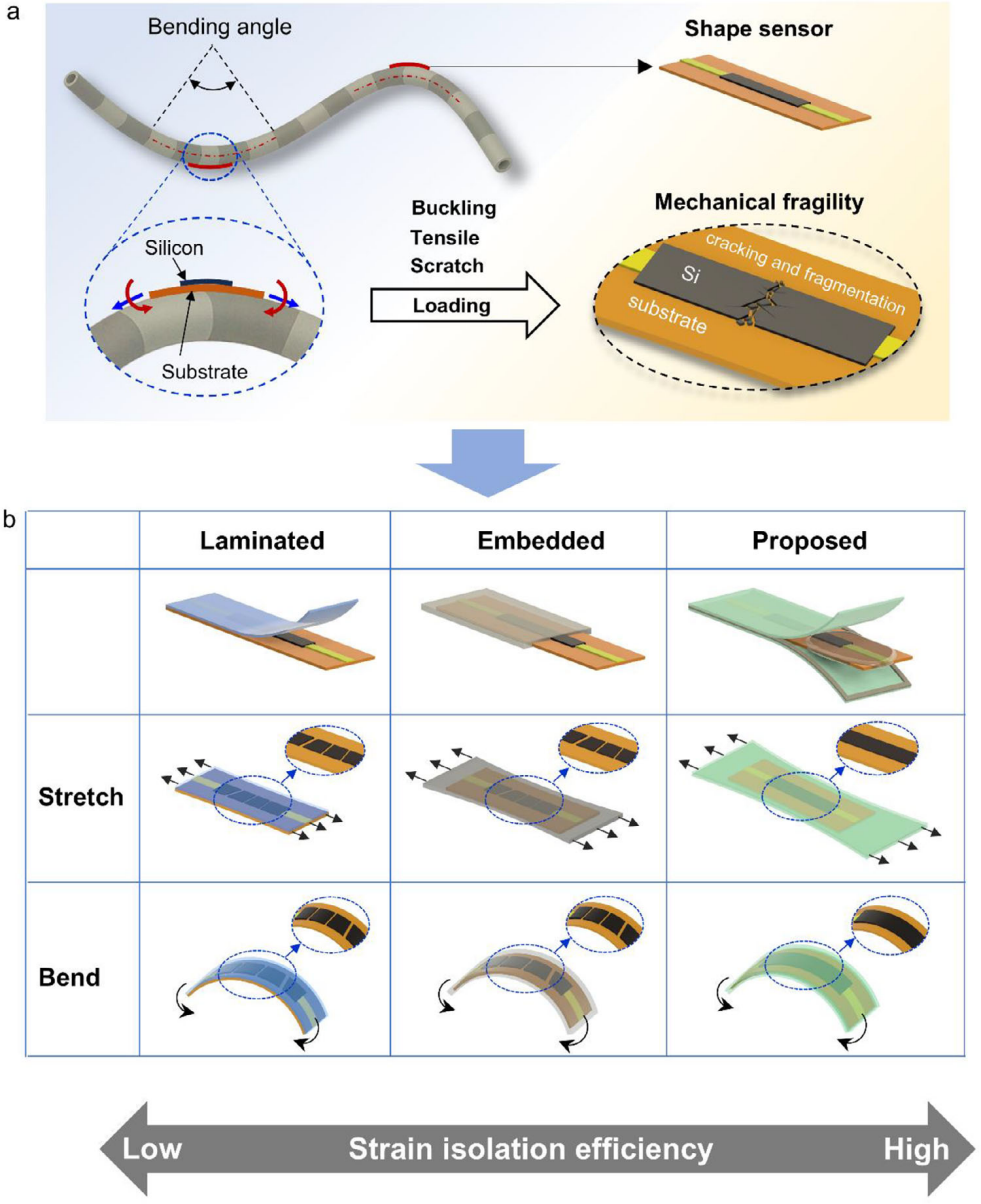
(a) Ultrathin Si‐based sensor used for shape sensing, which requires protective packaging because of its mechanical fragility. (b) Comparison of three packaging strategies—laminated, embedded, and the proposed design—highlighting the enhanced stress isolation achieved with the proposed approach.

Many types of sensors have been developed to measure deformation in soft structures, including optical‐based [9], magnetic‐based [10], and soft functional material‐based sensors [11]. However, optical fiber sensors are difficult to integrate onto polymer surfaces, and their detection systems are typically bulky and costly for medical robotic applications [9]. Magnetic‐based sensors require external field‐sensing units positioned around the robot, complicating system design and limiting miniaturization [10]. Soft polymer sensors are promising but suffer from reduced long‐term reliability because viscoelastic effects lead to sensitivity drift over time [11]. An ultrathin inorganic silicon (Si) semiconductor–based shape sensor is therefore an appealing alternative because it lacks viscoelastic behavior, enabling superior durability, sensing accuracy, and long‐term response stability [12]. However, Si sensors are highly vulnerable to even moderate stretching [13], surface scratching [14], and compression‐induced buckling [15], as external strain is transferred almost entirely to the brittle Si layer [16, 17]. This issue is especially severe in ultrathin Si, which typically fractures below 1 % strain [18], making it extremely fragile under bending, tension, or impact loading [19, 20], as illustrated in Figure 1a. Therefore, robotic skins with shape sensors—including both organic and inorganic thin‐film sensors—are typically encapsulated in thick elastomers or polymer films to shield the fragile sensing elements from external tensile and compressive stresses. Developing a packaging strategy that offers robust mechanical protection without degrading sensing performance is essential for the practical implementation of shape sensors. As shown in Figure 1b, two commonly used packaging approaches are laminated and embedded structures [21, 22]. Laminated designs typically involve bonding polymer films such as polyimide (PI) [23], polyethylene terephthalate (PET) [24], or polyethylene naphthalate (PEN) [25] onto the sensing element using adhesives, thermal lamination, or applied pressure. However, these layers often transmit external stress directly to the thin‐film sensor, rendering ultrathin Si highly vulnerable to mechanical fracture during tensile or bending deformation [26, 27]. Embedded designs, which use soft elastomeric materials such as polydimethylsiloxane (PDMS) [28], Ecoflex [29], or hydrogels [30], provide improved strain buffering but still suffer from interfacial delamination and long‐term material degradation [31, 32]. Although these approaches attempt to position the sensing element near the packaging's neutral axis to reduce bending‐induced strain [33], achieving accurate alignment on the curved, deformable surfaces of soft medical robots is challenging, and tensile strain remains largely unmitigated. Consequently, conventional packaging strategies cannot effectively decouple tensile and bending deformations.

To address these limitations, we propose an oil‐lubricated slidable (OLS) packaging strategy for 5 µm‐thick Si‐based sensing elements. The design employs a dual‐layer flexible film filled with silicone oil, enabling the sensing element to slide freely within the encapsulation and thereby achieving near‐zero strain transfer under external mechanical loading. Compared with conventional laminated and embedded packaging approaches, this structure effectively protects the device from tensile and compressive stresses while preserving sensing accuracy and response stability. Using the OLS structure in combination with a stretchable hot‐melt polyurethane sheet (HMS) film, the packaged sensor provides accurate and reliable bending detection even under large applied strains. It demonstrates high dynamic measurement accuracy, fast response, low hysteresis, and excellent durability, underscoring its engineering practicality and offering a promising approach for developing stretchable shape sensors. The proposed structure has also been integrated into a flexible endoscope for real‐time 360° bending detection, highlighting its potential for robust packaging and reliable operation in complex 3D deformation environments.

## 2 Results and Discussion

### 2.1 Structure, Fabrication, and Sensing Mechanism

**FIGURE 2:**
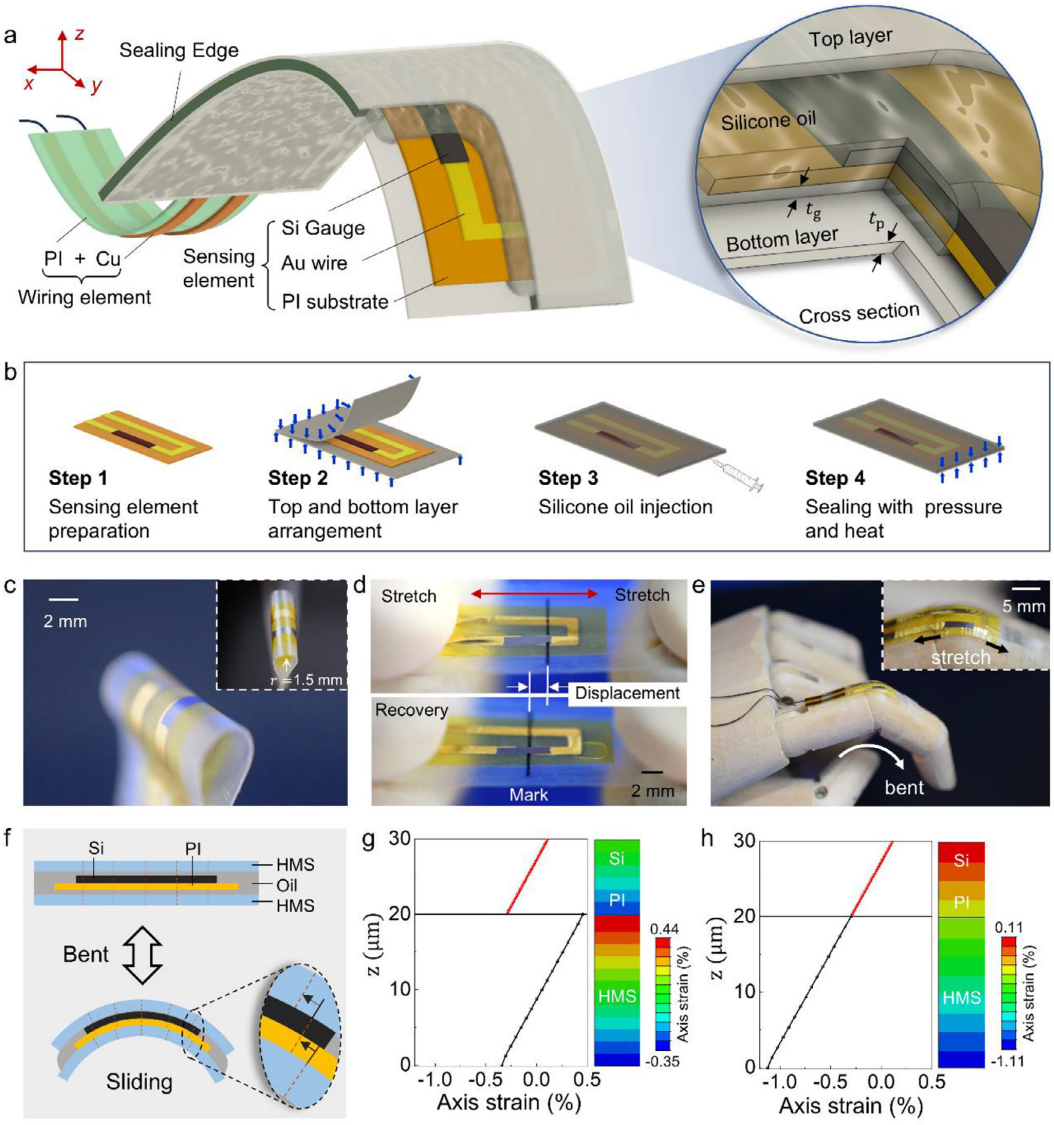
Structure, fabrication, and sensing principle of the OLS‐packaged sensor. (a) Schematic illustration of the proposed packaging architecture and corresponding cross‐sectional details. (b) Fabrication workflow of the packaging structure. (c) Photograph of the OLS‐packaged sensor integrated with the HMS. (d) Photograph of the OLS‐packaged sensor under tensile loading, showing the Si‐based sensing element sliding freely within the package. (e) Photograph of the OLS‐packaged sensor mounted on a finger model during bending. (f) Schematic depiction of the interlayer sliding mechanism. (g) Axial strain distribution across the thickness direction for the proposed packaging structure at a bending radius of 2.5 mm. (h) Axial strain distribution for a conventional packaging structure under the same bending condition.

Figure 2a illustrates the proposed OLS packaging structure for a 5 µm‐thick Si gauge (fabricated following previously reported processes [34, 35] bonded onto a 5 µm‐thick PI substrate (Xenomax, Toyobo). The packaging consists of two thin polymer films of equal thickness that are sealed along the edges and filled with a low‐viscosity silicone oil. The ultrathin sensing element is fully immersed in the oil, as shown in the magnified cross‐sectional view in Figure 2a. Owing to the fluidity of the silicone oil (KF‐96‐100CS, Shin‐Etsu Chemical Co.), extremely thin lubrication layers form between Si and the top film and between the PI substrate and the bottom film, thereby minimizing friction at both interfaces. One end of the assembled sensor is fixed to the packaging structure, while the opposite end remains free. Detailed overall and cross‐sectional dimensions are provided in Figure S1.

Fabrication involves preparation of the sensing element (Step 1) followed by the packaging process (Steps 2–4), as shown in Figure 2b. The sensing element, consisting of an ultrathin piezoresistive Si gauge and a PI substrate with Au wiring, was fabricated following previously reported procedures (Figure S2) [34]. Briefly, the Si gauge was prepared from a Si‐on‐insulator (SOI) wafer using standard Microelectromechanical Systems techniques, including ion implantation, annealing, and etching [35]. The gauge was then released and bonded to the PI substrate via water‐vapor plasma‐assisted bonding; the PI substrate contained Au wiring deposited by sputtering [36]. In Step 2 (HMS packaging layer arrangement), the sensing element was positioned between the packaging films, which were thermally fused along three edges to hold the structure in place. In Step 3, a calibrated syringe was used to inject a precisely controlled volume of silicone oil. Finally, in Step 4, thermal pressure was applied to seal the remaining edge, producing a fully enclosed and leak‐free encapsulation.

Detailed experimental parameters and additional information for each fabrication step are provided in the Experimental Section. Figure 2c shows photographs of the OLS‐packaged sensors encapsulated with HMS films. As shown in the inset, the entire packaged sensor conforms smoothly to a bending radius of approximately 1.5 mm without damage, demonstrating excellent mechanical flexibility. Figure 2d further shows that the OLS‐packaged sensor can be easily stretched by hand. During deformation, the markers on the HMS film display clear displacement, indicating external strain, while the ultrathin Si‐based sensing element remains stationary throughout stretching and recovery. This decoupling occurs because the lubricating silicone oil allows the sensing element to slide freely within the package. Figure 2e illustrates the OLS‐packaged sensor integrated onto the finger of a hand model. During finger bending, the structure experiences complex deformation involving simultaneous stretching and bending, yet the sensor maintains accurate measurement performance. These results highlight the broad adaptability and robustness of the proposed OLS packaging method under diverse mechanical loading conditions.

Figure 2f illustrates the working mechanism of the OLS‐packaged sensor. The packaging forms a sealed cavity using top and bottom stretchable HMS films, fully immersing the Si‐based sensing element in a low‐viscosity silicone oil. The primary objective of this design is to isolate the Si‐based sensing element from axial strain transmitted through the packaging layers during operation, while still allowing it to bend freely. Bending‐induced deformation is detected through the piezoresistive response of the ultrathin N‐type doped Si layer, which functions as the strain‐sensitive element and enables high‐fidelity curvature measurement. The fluidity of the silicone oil allows the sensing element to slide relative to the packaging films, creating a “sliding‐to‐mechanical‐isolation” mechanism that effectively suppresses strain coupling typically observed in conventional multilayer composite structures. To evaluate the mechanical decoupling performance of the OLS package, a 2D finite element model (FEM) was developed.

Particular attention was given to comparing the strain experienced by the encapsulation films with that of the sensing layer, enabling a quantitative assessment of how effectively the OLS package isolates the sensing element from externally applied deformation. As shown in Figure 2g, the axial strain distribution along the thickness direction was obtained from FEM simulations under a bending radius of 2.5 mm with a 20 µm‐thick HMS packaging film. A bending radius of 2.5 mm was selected because it represents an extreme but realistic curvature encountered in endoscopic and soft robotic applications, while remaining within the mechanical tolerance of the ultrathin Si‐based sensor. In the strain profile, the black curve corresponds to the packaging film, and the red segment denotes the sensing element. The results reveal a distinct discontinuity in axial strain within the top and bottom packaging layers and at their interfaces with the sensing element, indicating effective mechanical decoupling. This behavior confirms effective mechanical decoupling, in which deformation of the outer films does not transfer directly to the sensing element. In contrast, Figure 2h shows the case where all layers are rigidly bonded. Here, the strain distribution is continuous across the entire thickness, indicating strong mechanical coupling. Under such conditions, external deformation applied to the packaging layers is transmitted to the sensing element, increasing the risk of signal distortion or mechanical damage. According to the Kirchhoff hypothesis [33], when layers are ideally bonded, the normal strain along the thickness direction (z‐direction) must remain continuous across the interface. This condition can be expressed as:

(1) $${\left[\varepsilon_{\mathrm{zz}}\right]}_{\mathrm{interface}}=0$$

The equation indicates that strain must remain continuous across material interfaces in perfectly bonded multilayer structures. Therefore, the strain discontinuity observed in our simulation verifies the strain‐isolation capability of the OLS packaging design. Together, the structural configuration and underlying working mechanism establish a robust sensing architecture that enables effective strain decoupling and high‐fidelity bending detection.

In practical applications, the device may experience incidental normal pressure during handling or operation, making it important to determine whether such loading affects the sensor response. To examine interfacial friction and stress‐transfer behavior under pressure, we performed additional sliding‐friction and electrical measurements using the setup shown in Figure S3. The results show that the dynamic friction coefficient decreases to ∼0.07 under high normal pressure, while the corresponding resistance change in the Si gauge remains as small as ∼0.2 %, indicating minimal shear‐induced tensile strain. These findings confirm that the sensing element, fully encapsulated within a sealed HMS cavity filled with silicone oil, consistently operates under an OLS interface. Because the confined silicone oil redistributes without escaping during compression, solid–solid contact is avoided, interfacial shear transfer is strongly suppressed, and out‐of‐plane pressure does not generate longitudinal strain in the sensing element. This sealed, OLS interface is the fundamental mechanism that decouples external pressure from the bending‐induced in‐plane deformation that the sensor is designed to detect.

### 2.2 Strain‐Isolation Behavior and Dynamic Response

**FIGURE 3:**
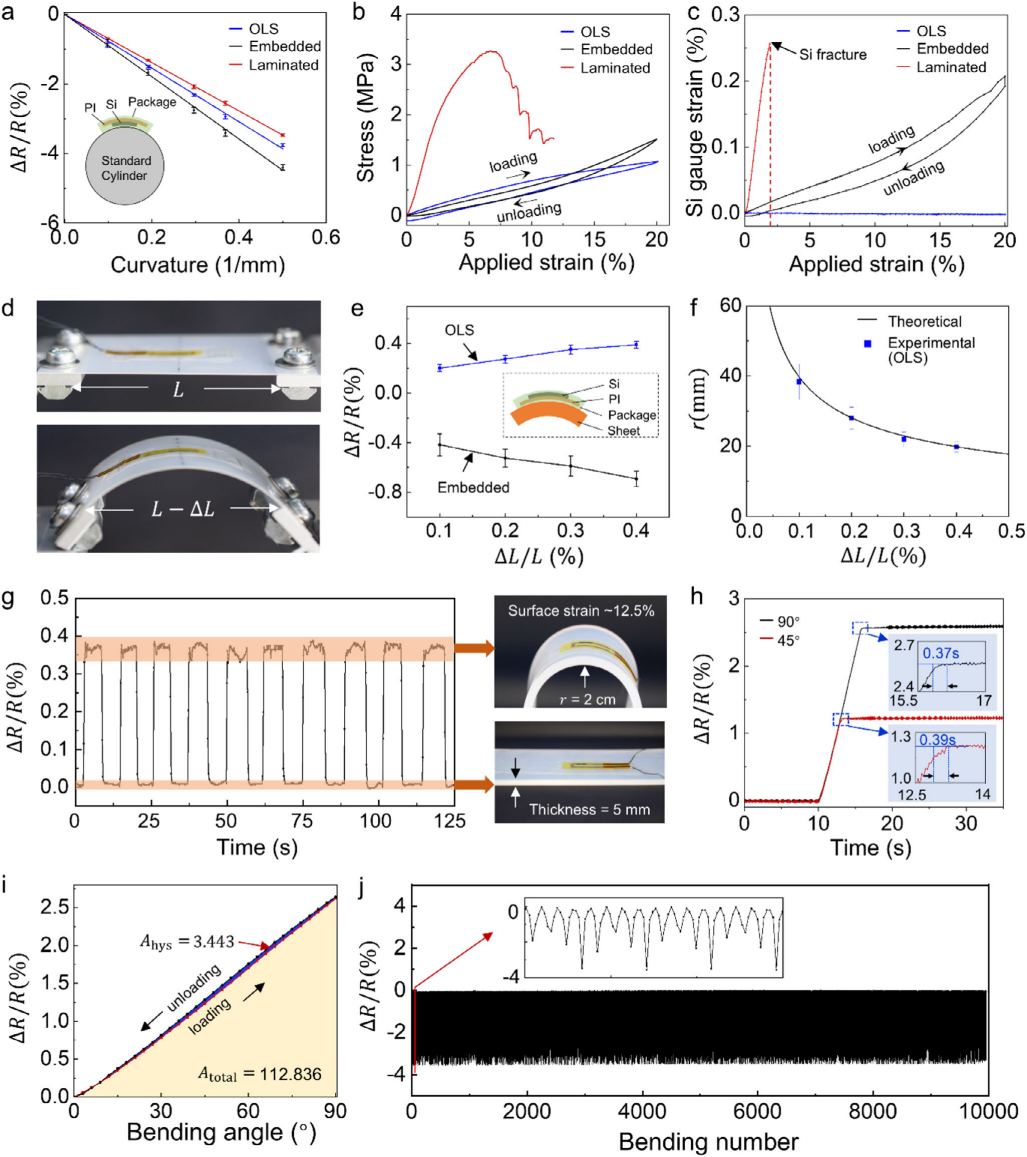
Performance evaluation of the OLS‐packaged sensor. (a) Comparison of resistance responses from laminated, embedded, and OLS‐packaged sensors under varying bending curvatures. (b) Stress–applied strain curve and (c) corresponding Si gauge strain–applied strain curves for sensors with laminated, embedded, and OLS packaging structure under an applied tensile strain up to 20 %. (d) Experimental setup showing the OLS‐packaged sensor attached to a 0.5 mm‐thick plastic sheet under bending. (e) Comparison of resistance changes between OLS and embedded packaged sensors. (f) Comparison of experimentally measured bending radii vs. theoretical predictions for the OLS‐packaged structure. (g) Dynamic bending of the OLS‐packaged sensor attached to a 5 mm‐thick rubber sheet. (h) Relative resistance changes for step bending from 0° to 45° and 90°. (i) Resistance response of the OLS‐packaged sensor during repeated bending and unbending between 0° and 90°. (j) Resistance response of the OLS‐packaged sensor during 10 000 bending cycles at a bending radius of 2 mm.

Three packaging strategies were fabricated and evaluated. The laminated packaging used a 30 µm‐thick Surgin film formed through thermal pressing. The embedded packaging was produced by spin‐coating PDMS to a thickness of 100 µm. The proposed OLS packaging utilized 50 µm‐thick HMS films sealed via thermal compression. Physical prototypes of the laminated and embedded sensors are shown in Figure S4. To compare bending sensitivity, all sensors were tested under a series of predefined curvatures, and the calibration results are presented in Figure 3a. For each curvature radius, five repeated measurements were conducted to obtain the sensing response. The sensitivity of a bending sensor is defined as the rate of change in resistance with respect to bending curvature, expressed as:

(2) $$S=\frac{\Delta R}{R_{0}}/\frac{1}{r}\;\left(1/mm^{-1}\right)\;$$

where Δ*R* is the resistance change, *R*_0_ is the initial resistance, and 𝑟 is the bending radius. The sensitivity represents the change in electrical resistance per unit change in curvature. The corresponding sensitivity values and coefficients of determination (*R* ^2^) are summarized in Table S1. All sensors exhibit a linear and stable resistance response to strain. In the laminated and embedded configurations, the packaging modifies the stiffness distribution and consequently shifts the neutral‐axis position, resulting in different effective distances between the piezoresistive layer and the neutral axis. This structural variation leads to the highest (embedded) and lowest (laminated) sensitivities among the three designs. In contrast, the OLS‐packaged sensor demonstrates a lower sensitivity (−0.077 (1/mm^−1^)), confirming its ability to mechanically isolate the sensing element. The sealed cavity and silicone‐oil‐mediated sliding interface suppress strain transfer from the packaging films, allowing the sensing element to experience minimal parasitic deformation and thus enabling more accurate bending detection. To assess the effectiveness of each packaging structure in shielding the Si gauge from external deformation during practical use, tensile tests were performed by applying macroscopic strain to the entire packaged device and quantifying the actual strain experienced by the Si gauge. Figure 3b presents the curves of applied strain vs. the corresponding gauge strain for the laminated, embedded, and OLS‐packaged sensors. For the laminated packaged sensor (red curve), the stress–strain response exhibits a distinctly brittle behavior. The applied stress rises sharply with increasing strain and reaches a peak at approximately 6 % strain, after which multiple abrupt drops occur. These discontinuities indicate local structural failures, such as interfacial delamination or crack propagation, during stretching. Such unstable behavior suggests that the laminated packaging suffers from poor mechanical reliability and is highly susceptible to stress concentration. Furthermore, the abrupt stress transfer demonstrates that this packaging strategy fails to effectively protect the ultrathin Si‐based sensing element from external mechanical loading.

As shown in Figure S5a, the Si element is highly susceptible to brittle fracture under tensile loading. In contrast, the embedded packaged sensor (Sylgard 184, Dow Corning) exhibits a gradual increase in slope beyond 12 % applied strain, indicating a strain‐dependent stiffening behavior. This response can be attributed to the thick PDMS encapsulation initially accommodating most of the deformation, whereas at higher strains the non‐stretchable PI substrate begins to bear a larger portion of the load, resulting in increased structural stiffness. A significant interfacial delamination between the stretchable PDMS layer and the PI substrate is also observed, as shown in Figure S5b. For the OLS‐packaged sensor, the stress–strain curve exhibits a more uniform and stable response.

Owing to the mechanically isolated nature of the proposed packaging structure, the strain applied to the entire device is primarily absorbed by the HMS film, exerting minimal influence on the Si‐based sensing element. Consequently, the overall tensile response is governed by the intrinsic mechanical behavior of the HMS material. The gradual decrease in the apparent elastic modulus observed during stretching is attributed to the nonlinear softening of the polymer or to molecular chain reorientation under large strain. The detailed deformation behavior under tensile loading is illustrated in Figure S5c. Furthermore, the strain transmitted to the Si gauge was quantified by converting the real‐time resistance changes into the corresponding gauge strain during tensile loading. The detailed procedure for converting the piezoresistive signal into strain is provided in the Supporting Information. Figure 3c presents the relationship between the applied tensile strain for the three packaging structures and the resulting Si gauge strain. For the OLS‐packaged sensor, almost no external strain was transferred to the sensing element. Even under tensile strains as high as 20 %, the strain experienced by the Si gauge remained negligible during both loading and unloading, as evidenced by the nearly zero resistance change. This confirms the excellent strain‐isolation capability of the OLS design. In contrast, the embedded packaged sensor exhibited a substantial gauge strain of up to ∼0.21 %, indicating high strain‐transfer efficiency and the inability of the embedded structure to effectively decouple the sensing element from global tensile deformation. Although PDMS is intrinsically stretchable, the measured resistance signal does not fully capture the overall tensile behavior of the packaged structure because the Si gauge itself undergoes only minimal deformation (far below the applied 20 % macroscopic strain). In contrast, the laminated packaged sensor exhibits characteristic brittle‐fracture behavior, with the Si gauge strain increasing during stretching and then failing abruptly at a strain of approximately ∼0.26 %. Combined with the deformation patterns shown in Figure S5, these results demonstrate that, under large applied strains, the proposed OLS packaging reliably suppresses external stress transfer and provides robust protection for the brittle Si gauge. Consequently, in practical scenarios where simultaneous tensile and bending deformations may occur, the strain‐mode coupling inherent to laminated and embedded packaging structures complicates the separation of different deformation components, thereby reducing measurement accuracy and increasing the risk of structural failure.

Furthermore, we attached the embedded and OLS‐packaged sensors to a relatively thick (0.5 mm) rigid plastic sheet to emulate a target surface undergoing in‐plane strain. As shown in Figure 3d, when the sheet transitions from a flat configuration to a buckled state, its effective length decreases from the initial value 𝐿 to 𝐿 − Δ𝐿. The corresponding resistance variations measured during this process are presented in Figure 3e. As Δ𝐿/𝐿 increases, the two packaging types exhibit opposite resistance trends. Each point reflects ten repeated measurements, and the error bars denote the standard deviation resulting from slight variations in initial placement and loading. Theoretically, during bending, the piezoresistive Si gauge should experience compressive strain, resulting in an increase in resistance. However, in the embedded sensor, the high strain‐transfer efficiency of the PDMS (KE‐1300, Shin‐Etsu Chemical) encapsulation leads to direct transmission of the sheet's surface strain to the Si gauge. Consequently, the gauge is subjected to tensile strain instead, producing a decrease in resistance and thereby invalidating the intended bending measurement. According to Euler buckling theory, the bending radius at the midpoint of the plastic sheet can be calculated using the following theoretical expression [37]:

(3) $$r=\frac{L}{2\pi }\;\sqrt{\frac{L}{\Delta L}}$$

As shown in Figure 3f, the bending radii extracted from the OLS sensor outputs closely match the theoretical values, further validating the structure's excellent strain‐isolation performance and high bending‐measurement accuracy. Nevertheless, for larger bending radii, the induced strain becomes small, making the measurements more sensitive to minor fluctuations and leading to slightly larger deviations.

In practical applications, bending sensors are typically mounted on the surfaces of flexible bodies, where they experience not only tensile deformation but also repeated loading–unloading cycles. To assess the dynamic behavior under such conditions, an OLS‐packaged sensor was attached to a thick rubber sheet (5 mm) and manually bent to a radius of approximately 2 cm—comparable to the curvature of a human joint (Figure 3g, right). This bending cycle was repeated multiple times. The results show that, in the bent state, the resistance remained stable at approximately 0.37 %, which agrees well with the value predicted from Equation (2) using a sensitivity of −0.077 (1/mm^−1^) at a bending radius of ∼20 mm.

Although the surface strain of the thick silicone sheet reached ∼12.5 %, the soft shape sensor captured the dynamic bending accurately and consistently. These results demonstrate the effectiveness of the OLS packaging structure in isolating the sensing element from external strain and maintaining accurate measurements under complex deformation conditions. While the silicone oil in the OLS package provides excellent strain isolation through mechanical buffering, its fluid nature may introduce response delays and hysteresis during loading and unloading. Therefore, evaluating the sensor's dynamic response and hysteresis characteristics is essential. As shown in Figure S6, a standardized bending test platform was employed to characterize these features. Figure 3h presents the corresponding dynamic response performance. The bending motion was driven by a motorized rotation stage operating at a constant speed of 16°/s, ensuring that the measured response reflected the intrinsic behavior of the sensor rather than the actuator dynamics. The OLS‐packaged sensor was bent to 45° and 90°, and the response time was defined as the interval between the end of the bending motion and the point at which the output signal reached its steady‐state average for each angle. Using this criterion, the response times for 45° and 90° bending were determined to be 0.37 and 0.39 s, respectively, demonstrating rapid and stable response characteristics. Detailed timing information is provided in Figure S7. To quantify the consistency of the sensor output during loading and unloading, the hysteresis ratio *H* was defined as the ratio of the area enclosed between the loading and unloading curves (*A*_hys_) to the area under the loading curve (*A*_total_), as expressed by:

(4) $$H=\frac{A_{\mathrm{hys}}}{A_{\mathrm{total}}}\;\times 100\;\%$$

For the OLS‐packaged sensor, the hysteresis ratio was substantially lower, at approximately 3 %, compared with the embedded structure (Figure 3i). This performance is considered excellent among flexible bending sensors and is sufficient for applications such as dynamic angle detection, structural deformation monitoring, and wearable sensing. Notably, both packaging structures exhibited an atypical hysteresis pattern, in which the unloading curve lay slightly above the loading curve. This behavior is likely attributable to the rapid elastic recoil of the encapsulation materials, combined with the high elastic modulus of the ultrathin Si‐based sensing element, which accelerates strain recovery during unloading and results in a corresponding reduction in resistance. Such behavior is commonly observed in flexible electronic devices and further emphasizes the effectiveness of the proposed OLS packaging strategy in enhancing dynamic response stability. To evaluate long‐term durability, the OLS‐packaged sensor was subjected to cyclic bending with a radius of 2 mm, as shown in Figure 3j. Despite this extreme curvature, the sensor maintained a highly stable resistance response over 10 000 consecutive loading–unloading cycles, with no observable drift or performance degradation. This exceptional durability under tight bending conditions highlights the mechanical robustness and long‐term reliability of the OLS packaging structure, even in demanding dynamic applications.

### 2.3 Shape Sensing in Endoscopic Devices

**FIGURE 4:**
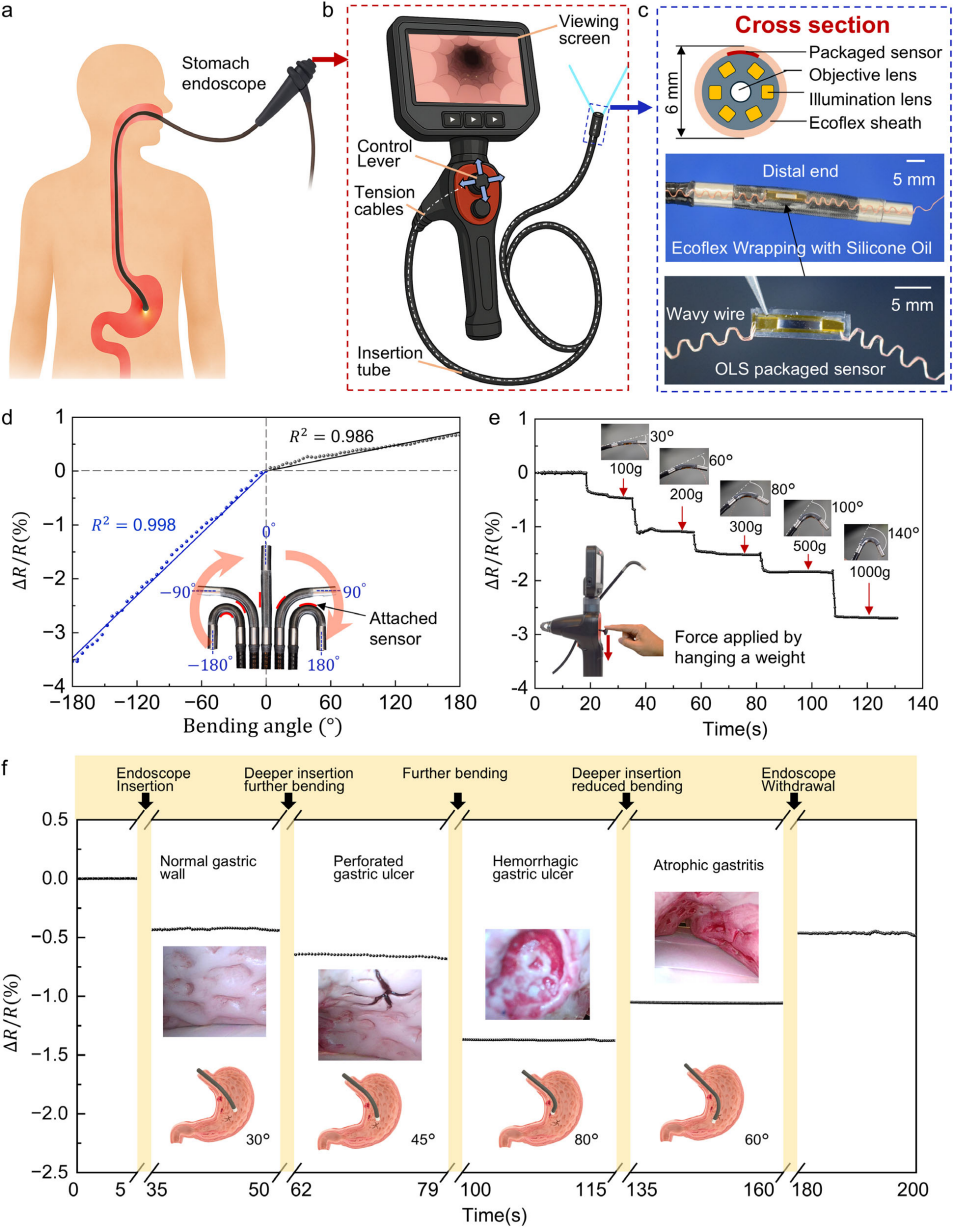
Integration and application of the OLS‐packaged sensor in a gastric endoscope system. (a) Simulation model of a gastric endoscopic examination scenario. (b) Structural diagram of a typical electronic gastroscope. (c) Integration details of the OLS‐packaged bending sensor at the distal end of the insertion tube. (d) Relationship between resistance and bending angle during unidirectional calibration from 0° to 360° at the distal end. (e) Correlation between the applied tensile force on the control lever and the resulting bending angle at the distal end. (f) Real‐time sensor response signal recorded during endoscopic navigation within a human stomach model.

Gastrointestinal endoscopy, particularly upper gastrointestinal endoscopy (gastroscopy), is among the most commonly performed clinical procedures (Figure 4a) [38]. However, most existing endoscopic devices cannot monitor real‐time shape changes of the insertion tube during navigation [39]. Physicians must therefore rely on visual feedback and manual experience, which makes it difficult to accurately perceive the spatial configuration of the endoscope within the body [40]. This challenge is especially pronounced in pediatric and elderly patients, whose gastrointestinal tracts are smaller and more fragile, increasing the risk of perforation or misinsertion. Thin gastroscopes, typically with outer diameters of 5–7 mm, provide enhanced flexibility but leave minimal space for integrating sensing components, creating significant design and operational constraints for safe and effective clinical use [41]. Achieving real‐time, high‐precision shape sensing in such flexible medical instruments is therefore crucial for improving procedural safety and navigation accuracy [42, 43]. Modern gastroscopy systems commonly used in clinical practice consist of three main components: the insertion tube, the control handle, and the visualization unit. The physician manipulates the control levers on the handle to actuate internal tension cables, which in turn drive the distal end of the insertion tube to bend in controllable up/down and left/right directions, allowing precise navigation and observation within the body (Figure 4b).

Leveraging this mechanical architecture, a gastroscope with a similar configuration was selected, and the proposed OLS‐packaged flexible bending sensor was integrated at the distal portion of the insertion tube to enable real‐time monitoring of local bending states. As illustrated in Figure 4c, the sensor was first encapsulated with an HMS thin film, and its signal lines were routed through a pre‐fabricated serpentine stretchable wire. A pre‐strained Ecoflex film was then applied as an outer layer to conformally attach the entire sensor assembly to the surface of the insertion tube (see the Experimental Methods section and Figure S8 for integration details). The final assembly maintained a total cross‐sectional diameter of approximately 6 mm, preserving sufficient flexibility and stable attachment without compromising the operational performance of the endoscope. This configuration effectively isolated the strain of the insertion tube body, enabling high‐sensitivity, low‐noise detection of local bending even in complex internal environments. For calibration, unidirectional bending of the distal end was performed incrementally over the full angular range from −180° to +180° (Figure 4d). The results demonstrated a strong linear correlation between the sensor's resistance change and the bending angle (*R* ^2^ ∼ 0.99), indicating excellent sensitivity and symmetric response, sufficient for precise angle perception required for comprehensive observation within the gastric cavity. Notably, when the sensor was positioned on the inner side of the bend, the bending radius was smaller (∼3 mm), resulting in a steeper slope, whereas on the outer side, the radius increased (∼10 mm), producing a shallower slope. These observations further confirm the wide measurement range and reliable linearity of the proposed packaged sensor across varying curvature conditions. To facilitate precise control of the insertion tube's bending by medical personnel or automated systems, a correlation between the applied operating force and the resulting bending angle was established. As shown in Figure 4e, different tensile forces were applied to the control lever by hanging calibrated weights, simulating manual input during endoscope operation. This procedure produced stable bending of the distal tip at discrete angles ranging from 30° to 140°. The sensor output exhibited clearly distinguishable signals for each bending state, demonstrating excellent stepwise resolution and repeatability. These results provide a reliable basis for quantifying operator input and could support intuitive force estimation for clinicians or enable closed‐loop control in robotic endoscopic systems.

Figure 4f presents the real‐time resistance signal recorded by an endoscope integrated with the OLS‐packaged ultrathin Si‐based sensor during navigation within a human stomach model. The monitored process included sequential stages: endoscope insertion, deeper advancement with increased bending, sustained bending, gradual reduction of bending angle, and final withdrawal. At each stage, the resistance variation (Δ*R*/*R*) accurately reflected the bending angle of the distal end of the endoscope—for example, approximately 30° during inspection of the normal gastric wall, ∼45° at the site of a perforated gastric ulcer, ∼80° near a hemorrhagic ulcer, and ∼60° at the region of atrophic gastritis. Transient fluctuations caused by hand‐induced vibration or momentary contact during insertion and adjustment were excluded to ensure the stability and reliability of the analyzed data. A detailed demonstration of the procedure is provided in Movie S1. By continuously monitoring the resistance signal of the shape sensor, the bending angle of the endoscope tip can be precisely determined. When combined with depth information from endoscope insertion, the spatial positions of gastric lesions can be accurately localized. The four regions shown in Figure 4f—normal tissue, perforated ulcer, hemorrhagic ulcer, and atrophic gastritis—correspond closely with both the resistance responses and endoscopic images, confirming the sensor's capability to provide reliable shape‐sensing feedback in complex anatomical environments. This system provides physicians with accurate, quantitative information, enhancing operational precision while reducing patient discomfort and procedural risk.

For practical applications involving contact with the human body, particularly during endoscopic procedures, the sensor must remain functional under elevated temperature and high humidity. To evaluate its electromechanical performance under such conditions, tests were conducted in a controlled environment with a relative humidity of 90 % and temperatures stabilized at 37°C and 45°C. Under each condition, 0°–90° bending tests were performed while continuously monitoring the resistance changes. As shown in Figure S9, the sensor maintained a highly linear and repeatable electromechanical response, demonstrating that high humidity and elevated temperature do not compromise its strain‐sensing capability.

Chemical robustness was further evaluated by immersing the packaged sensor in artificial sweat and an acidic solution (pH ≈ 1) at 37°C. During 23 min exposures, the real‐time resistance signals remained stable with negligible drift (Figure S10a,b), and subsequent 0°–90° bending tests showed no discernible loss of sensitivity or linearity (Figure S10c). These results confirm that the device can withstand both acidic and saline environments. Overall, the sensor demonstrates strong environmental stability under conditions relevant to human‐body applications, with any temperature‐induced signal variations being reversible and readily compensable during practical use. Detailed experimental procedures are provided in the Experimental Section.

## 3 Conclusion

In summary, this study presents an OLS packaging strategy for high‐fidelity shape sensing in soft medical robots. By introducing a lubricating silicone oil layer between stretchable HMS films, the ultrathin Si‐based sensing element is mechanically isolated from tensile and compressive stresses, effectively decoupling bending and tensile deformations. Unlike conventional laminated and embedded encapsulation, which require precise positioning of the sensor at the neutral axis, the OLS design removes this constraint while providing robust structural protection for fragile Si devices. This packaging approach enables accurate curvature detection even under 20 % applied tensile strain and achieves rapid response (<0.40 s), low hysteresis (∼3 %), and exceptional durability over more than 10 000 bending cycles at a tight radius of 2 mm. These results highlight the practical robustness and engineering applicability of the OLS structure for reliable sensing under complex mechanical loading. Moreover, successful integration into a flexible gastroscope demonstrates significant clinical relevance, enabling real‐time and stable shape sensing under continuous 360° bending—an essential capability for advancing flexible endoscopic robotics. This functionality establishes a foundation for intelligent features such as shape perception and force feedback in minimally invasive procedures, ultimately enhancing surgical safety and precision.

## 4 Experimental Section

### 4.1 Fabrication of the Sensing Element

The sensing element used in this study—including the ultrathin Si gauge, PI substrate with Au wiring, and bonding process—was fabricated following previously reported procedures [34]. Briefly, the Si gauge was microfabricated from an SOI wafer using ion implantation, rapid thermal annealing, and double‐sided dry etching to define the piezoresistive region and freestanding structure. Gold electrodes were then deposited via sputtering and patterned on a PI film, which served as the flexible substrate. The Si gauge and PI substrate were subsequently bonded using a water‐vapor plasma‐assisted Au‐Au bonding process, followed by transfer using water‐soluble tape to finalize the sensing element [36]. A schematic of the fabrication workflow is presented in Figure S2.

### 4.2 Preparation of OLS Packaging Structure

The prepared sensing element was first electrically connected to the copper traces of a flexible printed circuit board (FPCB) using anisotropic conductive film (ACF) tape (3M^TM^ 9703, 3M Company). The gold‐coated end was bonded under a pressure of 3 MPa at 150°C for 10 s to ensure reliable electrical contact. For the OLS‐packaged structure using HMS films, the top and bottom HMS layers were aligned and thermally bonded along three sides under a pressure of 0.5 MPa at 100°C for 15 s. Approximately 1‐µL volume of silicone oil (KF‐96‐100CS, Shin‐Etsu) was then dispensed into the cavity using a microliter syringe, and the final edge was sealed under the same temperature and pressure conditions to complete encapsulation. Each package was optically inspected to confirm complete sealing and the absence of air bubbles.

### 4.3 Preparation of Laminated and Embedded Packaging Structure

A medical polyurethane adhesive film (Kenz Surgin Film 30 µm, No.50, Suzuken Co., Ltd.) was used to cover the silicon side, ensuring full packaging with the device region. The film was then laminated by hot‐pressing at 80°C and 0.3 MPa for 1 min, forming a conformal laminated packaging structure. The embedded packaging structure was fabricated on a glass substrate by first spin‐coating a PDMS layer and then placing the Si‐based sensing element onto the partially cured surface, followed by spin‐coating another PDMS layer to fully embed the device. Two silicone elastomers were used for the encapsulation: a relatively stiff PDMS (Sylgard 184, Dow Corning; 10:1 base‐to‐curing‐agent ratio) and a softer RTV silicone (KE‐1300, Shin‐Etsu Chemical; Part A: B = 1:1). Each elastomer was mixed, degassed, and spin‐coated under the specified conditions to form ∼50 µm layers, followed by thermal curing.

### 4.4 Performance Characterization

Calibration was performed using stainless pin gauges with radii ranging from 2 to 10 mm (TEP Series, Trusco Nakayama Co.). Resistance changes were measured using an LCR meter (LCR‐6200, Good Will Instrument Co., Ltd.). Tensile experiments and additional friction tests under normal pressure were conducted with a programmable testing machine (FTN1‐13A, Aikoh Engineering Co., Ltd.). Angular motions for evaluating response delay and hysteresis were controlled using a motorized rotation stage (yaw axis, Model OS MS‐60YAW, SIGMAKOKI Co., Ltd.). Cyclic bending tests were carried out with a tabletop endurance tester (DLDM111LHA, Yuasa System Co., Ltd.). Corresponding resistance changes during these tests were recorded using a source meter (Keithley 2400, Tektronix Keithley Co., Ltd.).

### 4.5 Finite Element Analysis

Finite element simulations were performed using Abaqus 2023 (Dassault Systèmes). A simplified 2D symmetric model was employed for the analysis. Frictionless hard contact was defined between the encapsulation films and the sensing element to replicate the lubricating effect of the silicone oil. Symmetric boundary conditions were applied at the model center (displacement = 0). An arc‐shaped displacement was imposed on the bottom surface of the structure to simulate bending to a radius of 2.5 mm. The encapsulation films, Si, and PI substrate were all modeled using plane stress elements (CPS4R).

### 4.6 Sensor Fabrication and Integration to Endoscope

The sensing element (9 mm × 1.3 mm) was bonded onto a 1 mm × 2 mm FPCB electrode using ACF tape under 3 MPa at 150°C for 10 s. A wavy wire was then soldered to the electrode for signal extraction. The entire assembly was packaged with silicone oil and sealed using HMS films via thermal compression (100°C, 0.5 MPa, 15 s). Finally, a 100 µm‐thick bi‐axially pre‐stretched Ecoflex film was applied to conformally attach and integrate the sensor onto the outer surface of the endoscope. A handheld industrial endoscope (Ralcam F606B, Japan Amazon) was used for gastroscopic navigation, and a 3D anatomical relief human stomach model (3B Scientific, Model K17), designed for medical training and pathological demonstration, served as the test environment.

### 4.7 Compatibility Testing in Human‐Body Environments

Environmental tests were conducted to evaluate the sensor's stability under conditions relevant to human‐body applications. Temperature–humidity measurements were performed in a controlled chamber (SH‐222, ESPEC Corp.) set at 90 % relative humidity, with temperatures maintained at 37°C and 45°C. The sensor was bent from 0° to 90° using a motorized rotation stage (OS MS‐60YAW, SIGMAKOKI Co., Ltd.), while resistance changes were recorded with a source meter (Keithley 2400, Tektronix Keithley Co., Ltd.). The packaged sensor was immersed in artificial sweat (pH 5.5, NaCl ∼5 g/L, MUTO PURE chemicals CO., LTD, No. 81301) and an acidic solution prepared from diluted hydrochloric acid (pH ≈ 1, 36.5 %, FUJIFILM Wako Pure Chemical Corporation, No. 080–01066). Both solutions were maintained at 37°C on a hot plate. Real‐time resistance signals were monitored continuously for 23 min, followed by 0°–90° bending tests using the same motorized rotation stage and source meter setup described previously.

## Conflicts of Interest

The authors declare no competing interests.

## Supporting information




**Supporting File 1**: advs74288‐sup‐0001‐SuppMat.docx.

**Supporting File 2**: advs74288‐sup‐0002‐SuppMovie.mp4.

## Data Availability

The data that support the findings of this study are available from the corresponding author upon reasonable request.
