# Supplementary material for: A Strain Decoupling Packaging Strategy for High‐Fidelity Ultrathin Silicon Shape Sensors for Soft Medical Robotics
Source: Adv Sci (Weinh). 2026 Feb 26;13(22):e18733. doi: 10.1002/advs.202518733 (PMC13088285; doi:10.1002/advs.202518733)
Supplement: Supplementary file 1 — Supporting File 1: advs74288‐sup‐0001‐SuppMat.docx. [file ADVS-13-e18733-s001.docx]

Supporting Information

**A Strain Decoupling Packaging Strategy for High-Fidelity Ultrathin Silicon Shape Sensors for Soft Medical Robotics**

*Hao Liu, Masahito Takakuwa, Michitaka Yamamoto, Yiwen Wang, Tomoyuki Yokota, Takao Someya, Toshihiro Itoh, and Seiichi Takamatsu**

* Seiichi Takamatsu.

**Email:** stakamatsu@binghamton.edu

**This PDF file includes the following:**

Figures S1 to S10

Table S1

**Other supporting materials for this manuscript include the following:**

Movie S1

**Details of the fabrication process.**

**Fabrication of the Si gauge:** The Si gauge was fabricated from a Si-on-insulator (SOI) wafer (KST World Corporation) comprising a 400-µm-thick handle Si layer, a 1-µm-thick SiO₂ insulating layer, and a 5-µm-thick lightly doped p-type Si device layer. Phosphorus ions were implanted into the device layer and subsequently annealed at 950 °C for 30 s using a rapid lamp-annealing process (AS One, AnnealSys) to form a 150-nm-thick piezoresistive layer with a doping concentration of ~10²⁰ atoms∙cm⁻³. Cr and Au layers were deposited by conventional sputtering (ULVAC), followed by photolithography and etching to define Au electrode pads. Both the front (device) and back (handle) sides were patterned using inductively coupled plasma reactive ion etching (ICP-RIE; MUC-21, SPP) to define the sensor’s structural features for integration with the plastic-scale model.^[S1]^ Subsequently, the SiO₂ insulating layer was removed using reactive ion etching (RIE; RIE-10NR, SAMCO) to release the Si gauge. The released gauge was retrieved with a vacuum chip mounter (MRS-850RD, Okuhara Electric Corporation), which employed optical detection, precise positioning, and vacuum-assisted adsorption to handle and transfer the device.^[S1]^

**Fabrication of the sensing element:** A 5‑µm-thick PI film (XENOMAX, Toyobo Co., Ltd.) mounted on a Si wafer served as the flexible substrate. Cr (20 nm) and Au (100 nm) layers were sputtered onto the PI surface through a shadow mask to define the electrodes. The Au electrode on the Si gauge and the Au wiring on the PI substrate were activated by water vapor plasma treatment (Aqua Plasma Cleaner AQ-500, Samco Inc.) at 100 W with a 12-sccm gas flow for 40 s. The Si gauge was temporarily transferred using water-soluble adhesive tape (Water-Soluble Wave Solder Tape 5414, 3M) to facilitate alignment. The activated Au surfaces were then brought into contact and lightly pressed at room temperature (~25 °C), followed by thermal treatment on a hot plate at 150 °C under 10-kPa pressure for 30 min to promote Au–Au bonding. Finally, the assembly was immersed in 60 °C water for 10 min to dissolve the tape and release the bonded sensing element. ^[S2]^

**Method for Converting Resistance Response to Strain of Si gauge**

In pure bending, the bending radius $r$ of a planar cross-section is expressed as^[S3]^

$r=\frac{t}{\varepsilon}$, (1)

where $\varepsilon$ is the strain in the piezoresistive layer of the Si gauge, and *t* is the distance between the neutral axis and the piezoresistive layer, which can be determined using classical laminate theory.^[S4]^ The neutral-axis location for a multilayer structure depends on the Young’s modulus and thickness of each material layer:

$y=\frac{\sum_{i=1}^{2} E_{i}d_{i}\left[ \left( \sum_{j=1}^{i} d_{j} \right)-\frac{d_{i}}{2} \right]}{\sum_{i=1}^{3} E_{i}d_{i}}$, (2)

where $E_{i}$ and $d_{i}$ are the Young’s modulus and thickness of the $i$-th layer, respectively, and the summation is taken from the bottom to the top layer of the laminate. In this context, the thicknesses of the Si gauge and substrate layer are denoted as $d_{1}$ and $d_{2}$​, with corresponding Young’s moduli $E_{1}$ and $E_{2}$​. The piezoresistive behavior of the Si layer follows: $\Delta R/R_{0}=K_{S}\varepsilon$, where $\Delta R$ is the change in resistance due to strain, $R_{0}$ is the resistance of the undeformed layer, and $K_{S}$ is the gauge factor of the Si sensor. Accordingly, $r$ can be expressed as inversely proportional to $\Delta R$:

$r=\frac{tK_{S}R_{0}}{\Delta R}$. (3)

The differences in sensitivity among the laminated, embedded, and OLS structures arise from shifts in the neutral-axis position induced by the respective packaging configurations.

Based on a simplified Si–PI composite model and material parameters—Young’s modulus of 180 GPa for the Si gauge and 9 GPa for the PI substrate, both with thicknesses of 5 µm—the neutral-axis position was calculated using equation (2) to be 7.26 µm. This places the piezoresistive Si layer 2.26 µm from the neutral axis. Using equation (3) and the experimentally measured sensitivity coefficients (**Table S1**), the gauge factor of the OLS-packaged sensor is determined to be 33.19.


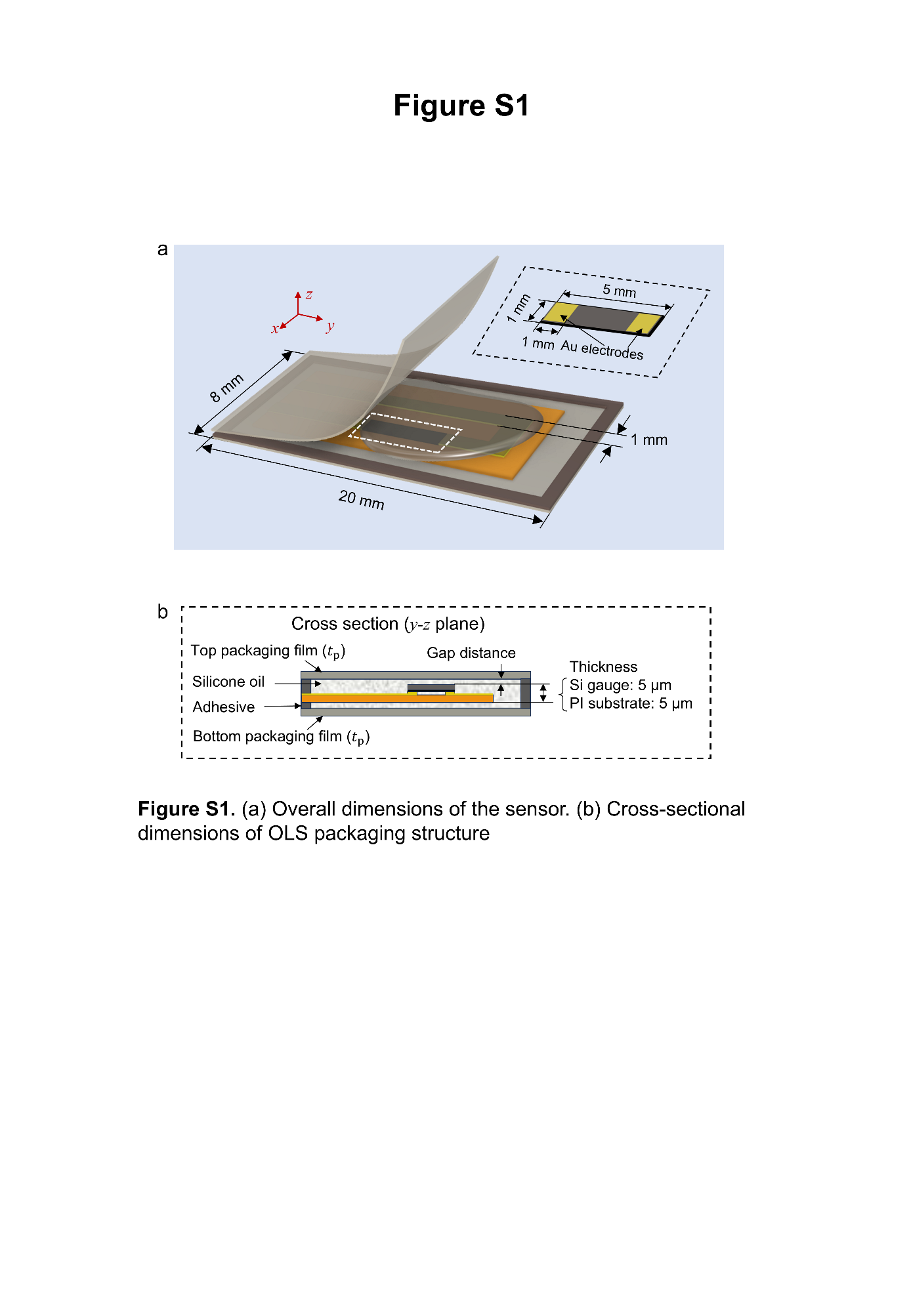


**Figure S1.** (a) Overall dimensions of the sensor. (b) Cross-sectional dimensions of the OLS packaging structure.


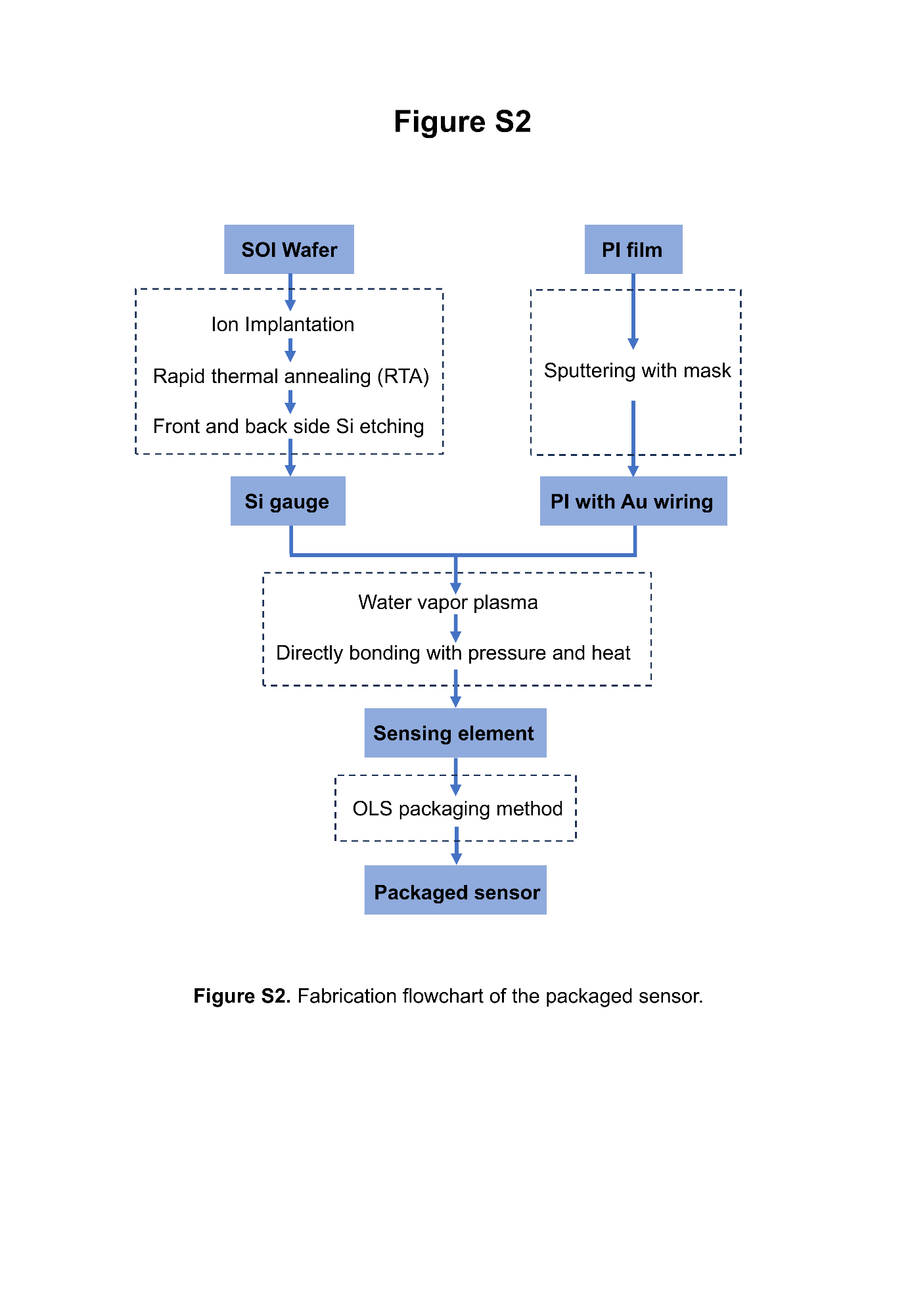


**Figure S2.** Fabrication flowchart of the packaged sensor.


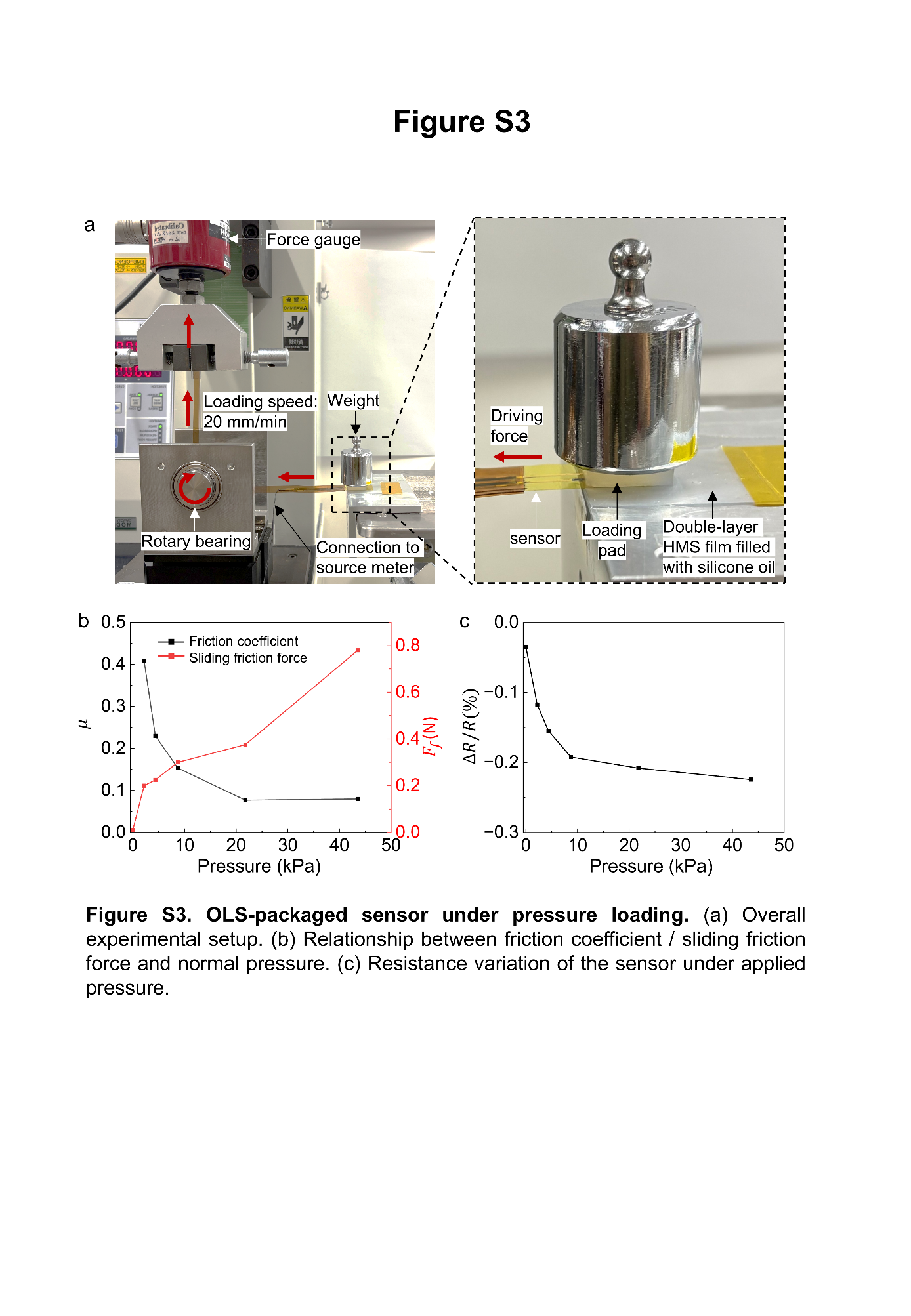


**Figure S3.** **OLS-packaged sensor under pressure loading.** (a) Overall experimental setup. (b) Relationship between friction coefficient/sliding friction force and normal pressure. (c) Resistance variation of the sensor under applied pressure.


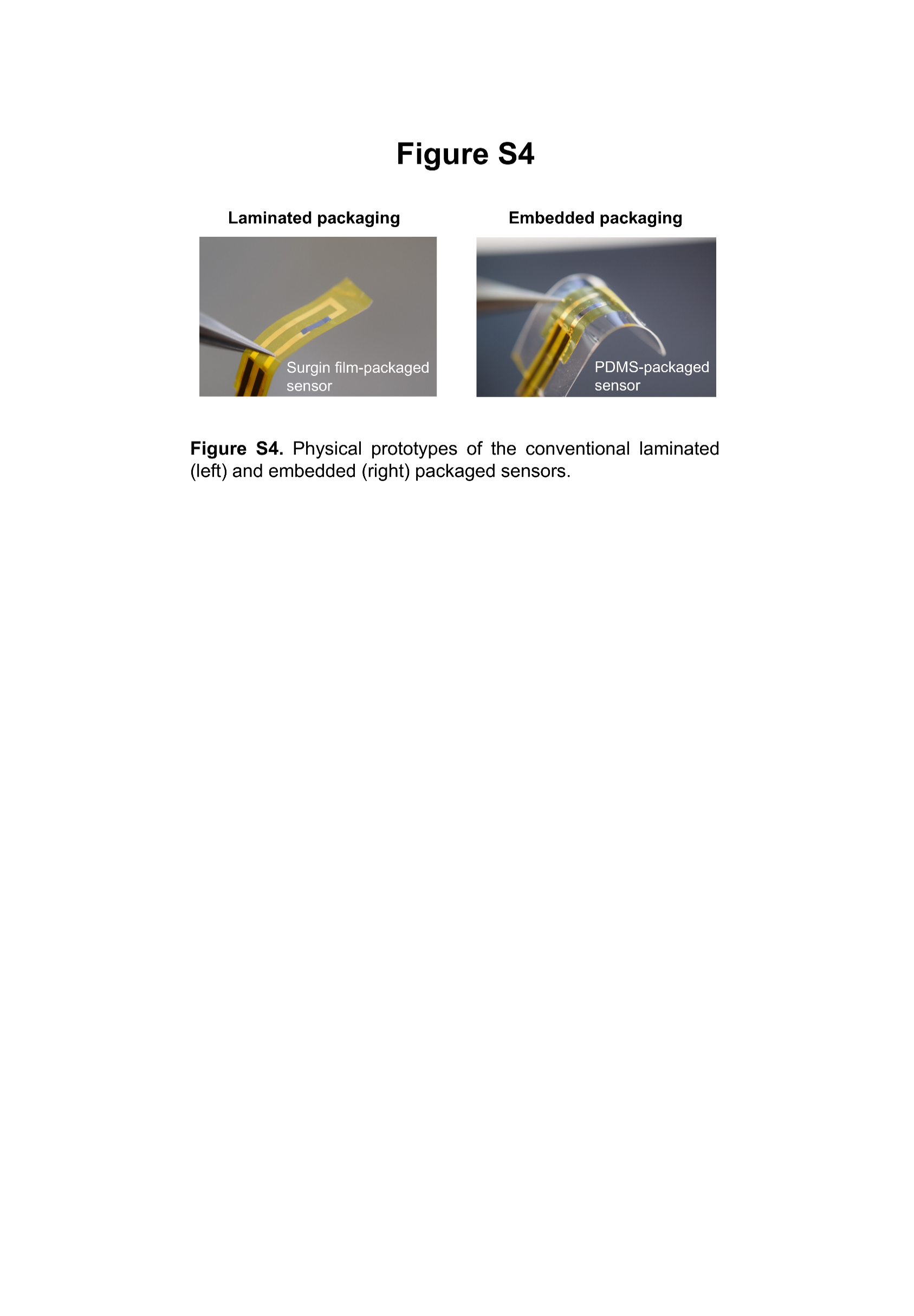


**Figure S4.** Physical prototypes of conventional laminated (left) and embedded (right) packaged sensors.

**Table S1.** Sensitivity and $R^{2}$ of sensors with laminated, embedded, and OLS packaging structures.

|  | Laminated | Embedded | OLS |
| --- | --- | --- | --- |
| Sensitivity | -0.069 | -0.090 | -0.077 |
| $R^{2}$ | 0.9994 | 0.9992 | 0.9992 |


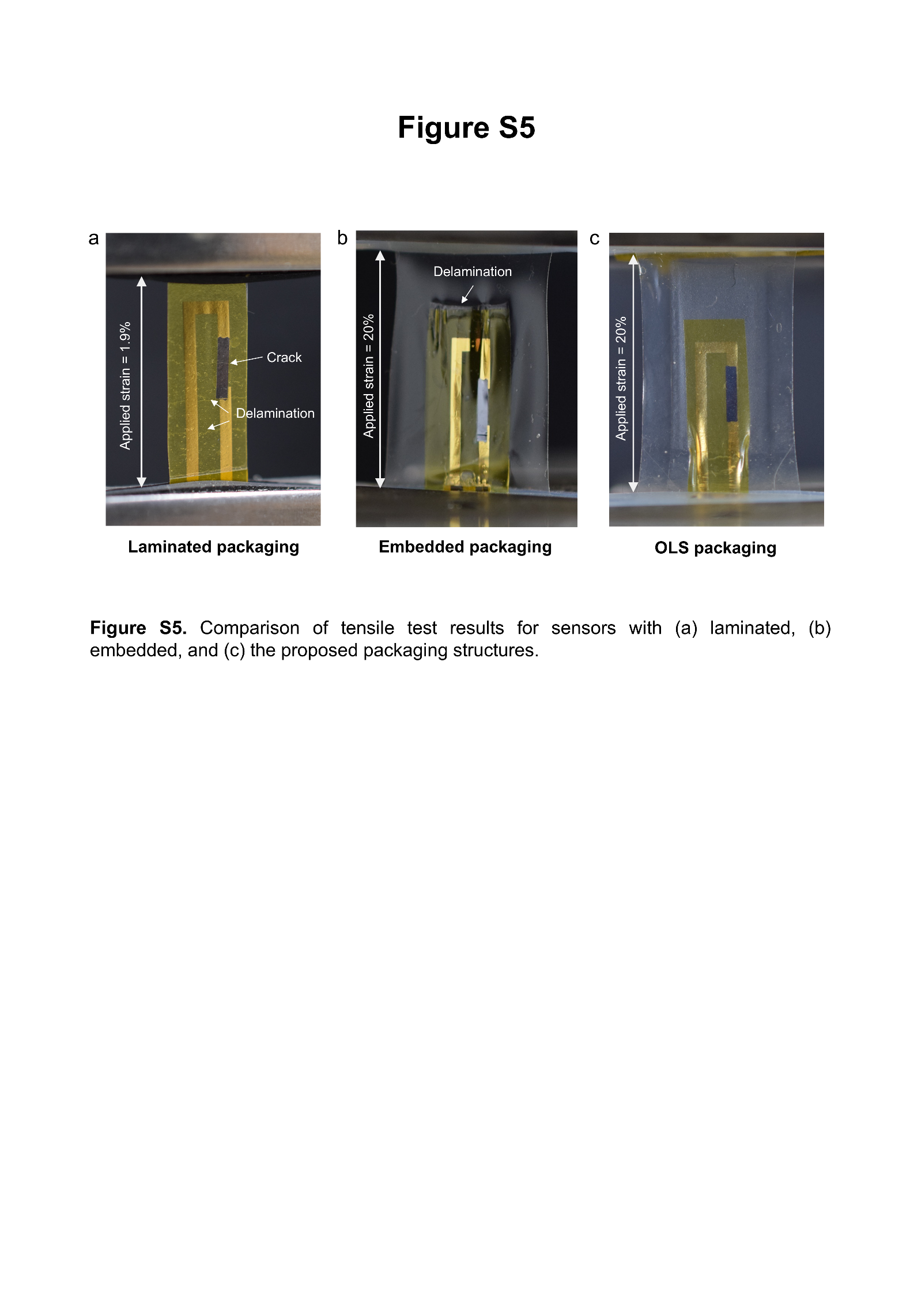


**Figure S5.** Comparison of tensile test results for sensors with (a) laminated, (b) embedded, and (c) the proposed OLS packaging structures.


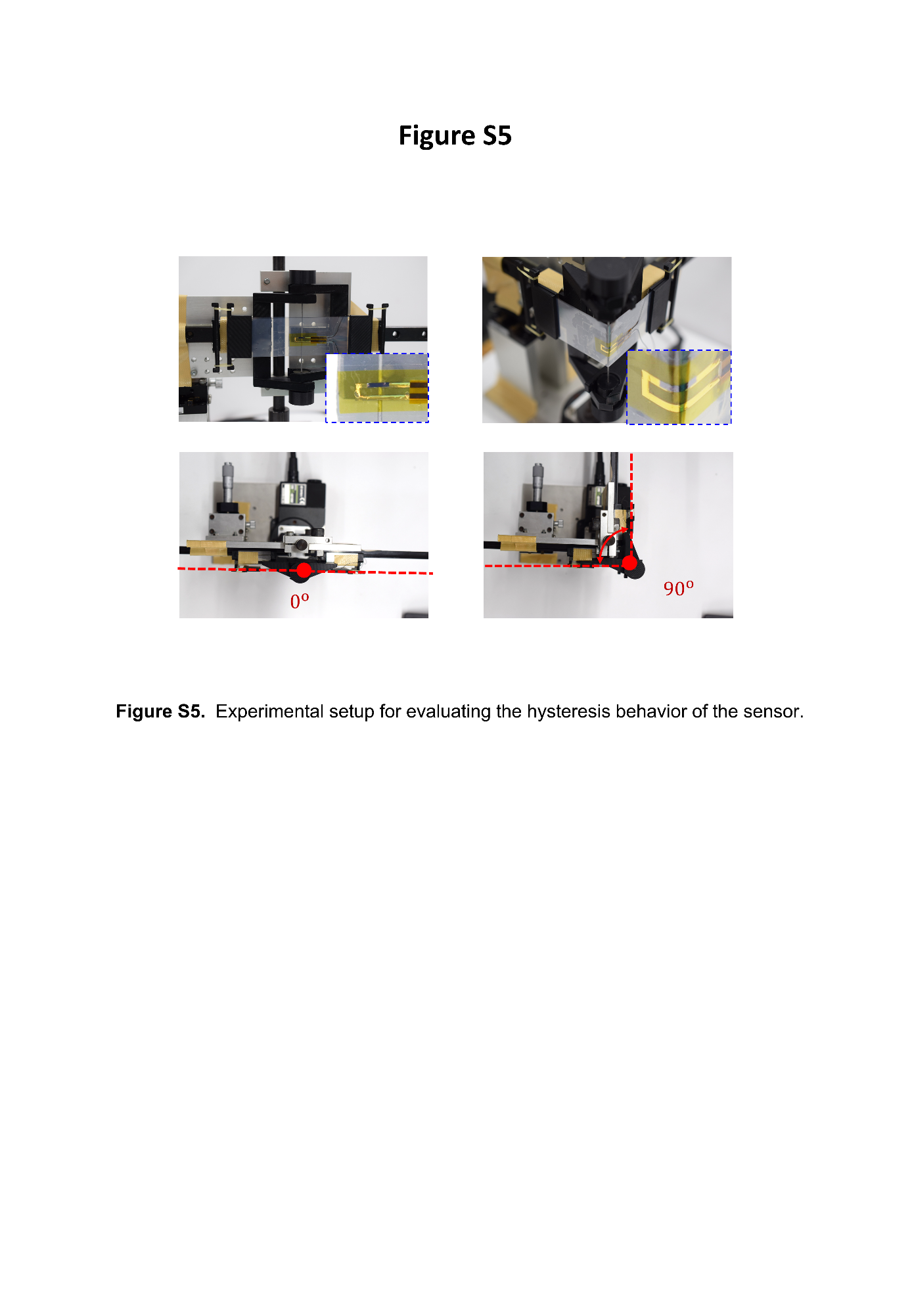


**Figure S6.** Experimental setup for evaluating the hysteresis behavior of the sensor.


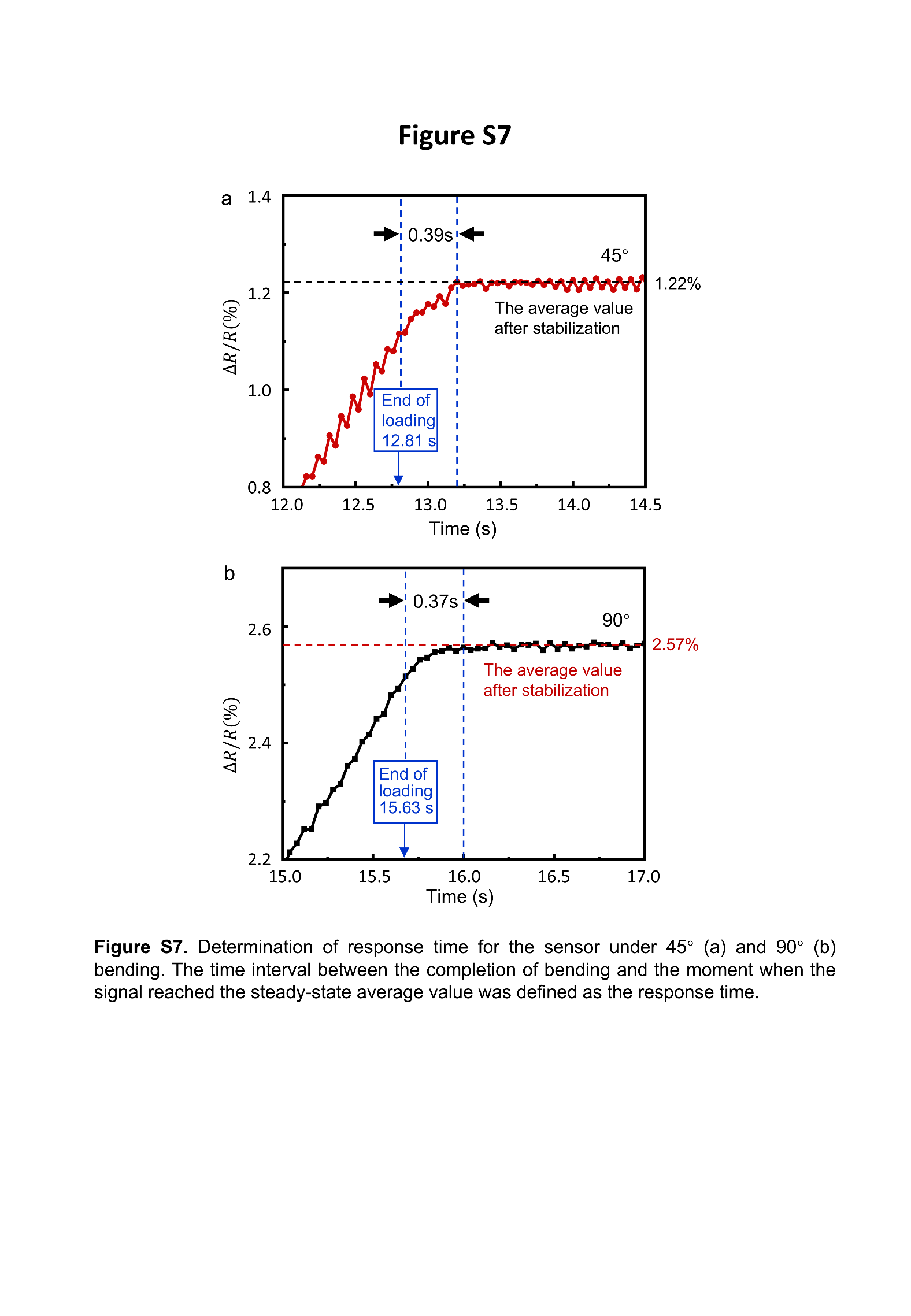
**Figure S7.** Determination of response time for the sensor under (a) 45° and (b) 90° bending. The response time is defined as the interval between the completion of bending and the moment when the signal reaches the steady-state average value.


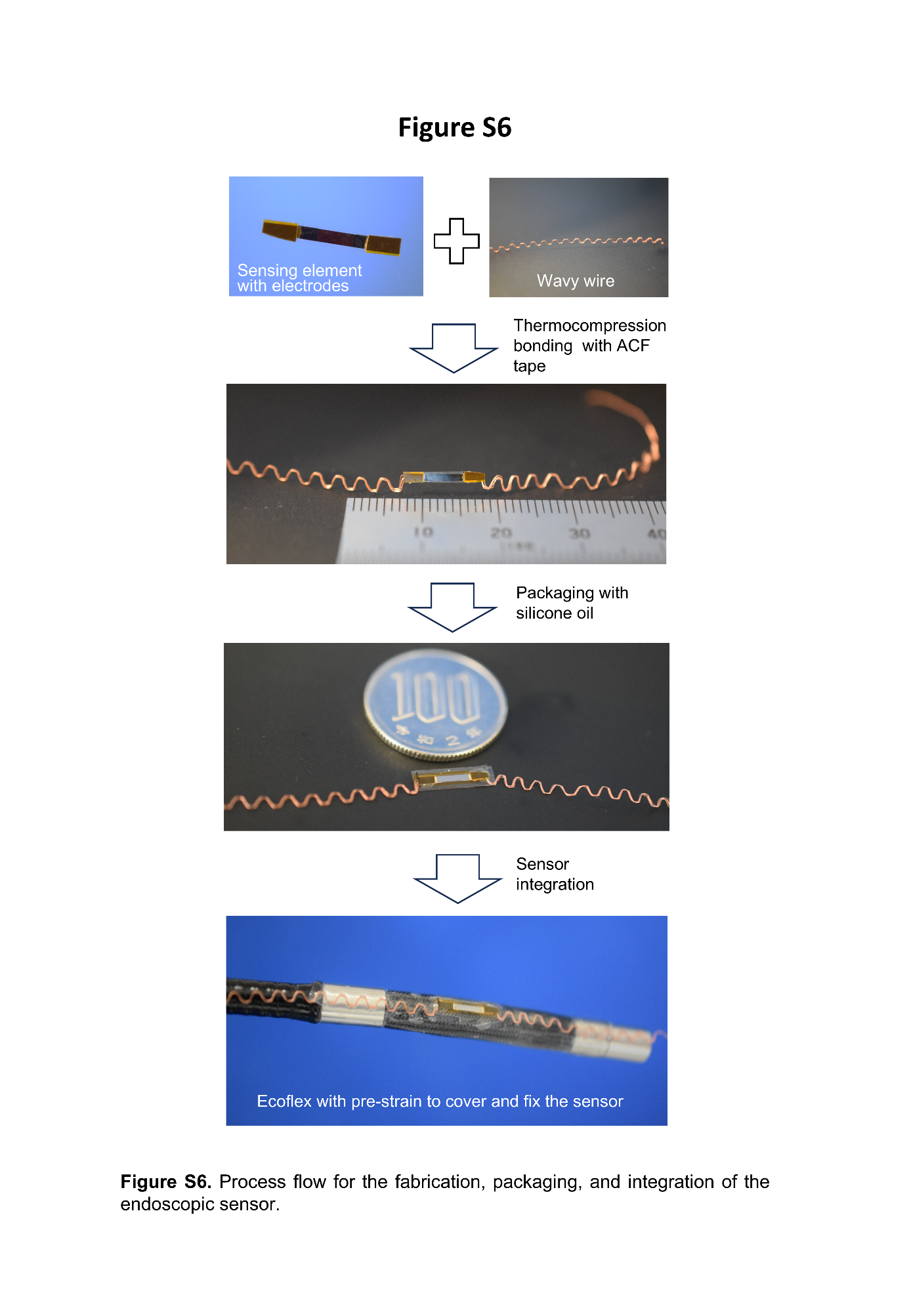


**Figure S8.** Process flow for the fabrication, packaging, and integration of the endoscopic sensor.


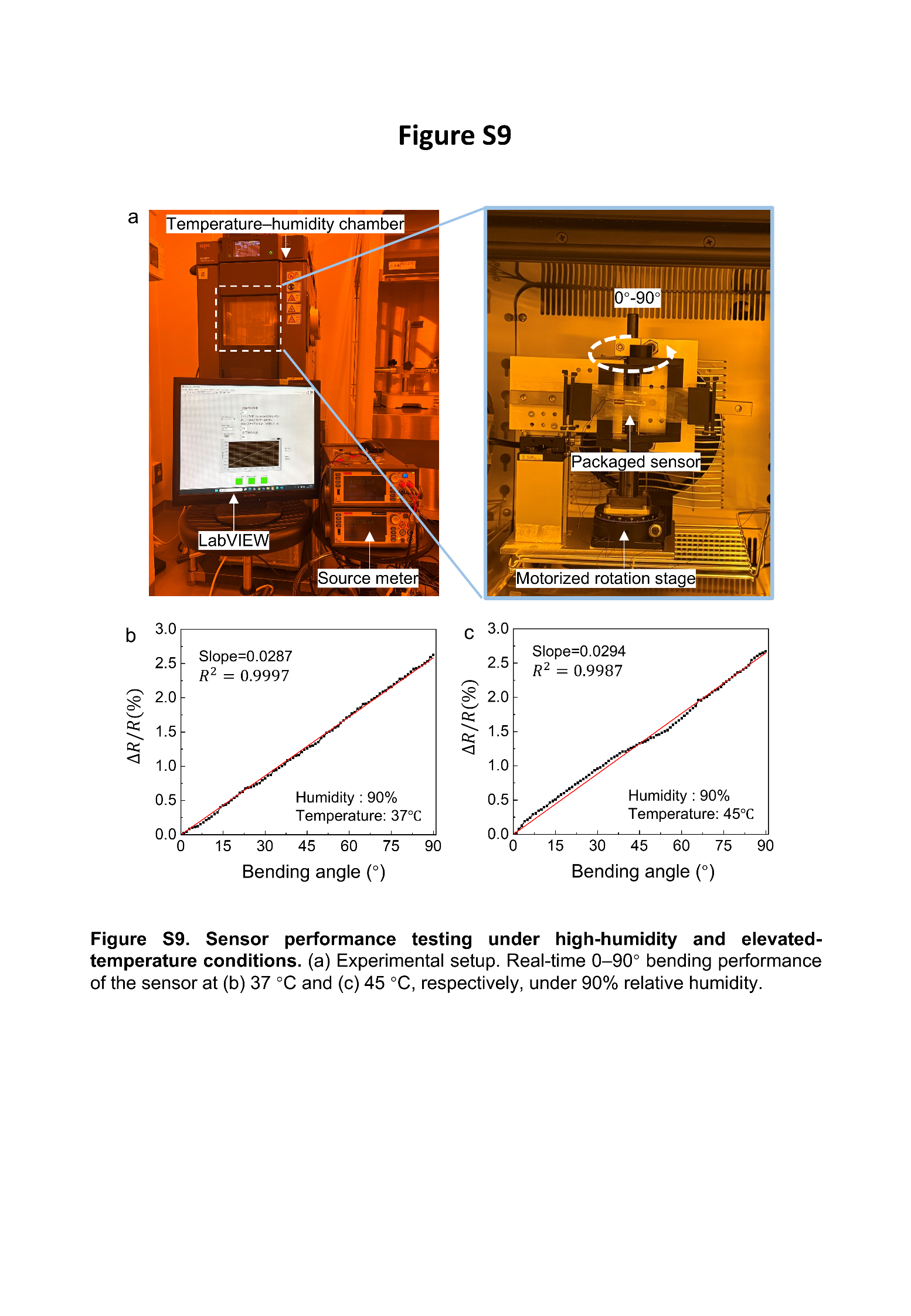
**Figure S9.** **Sensor performance testing under high-humidity and elevated-temperature conditions.** (a) Experimental setup. Real-time 0°–90° bending performance of the sensor at (b) 37 °C and (c) 45 °C under 90% relative humidity.


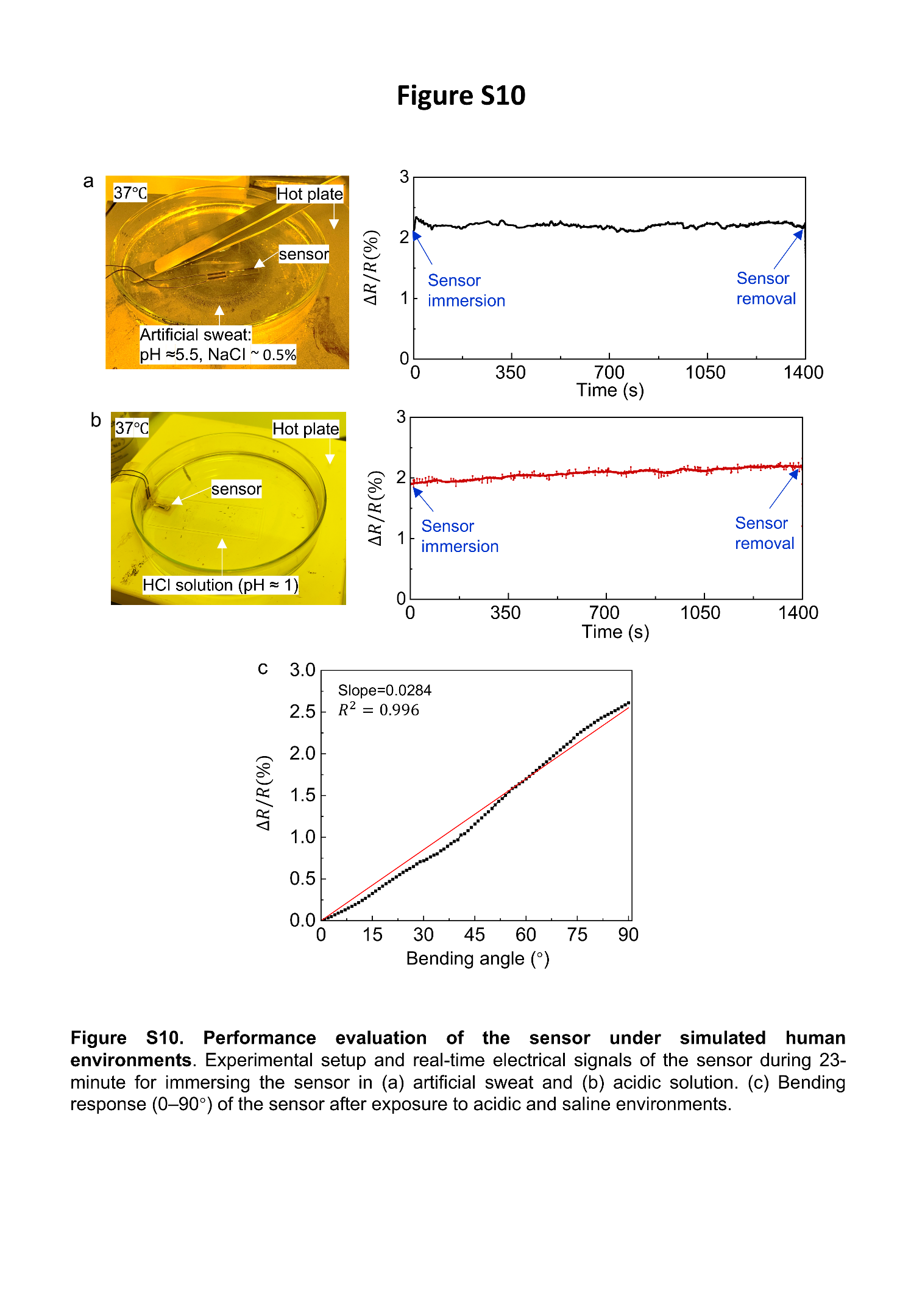
**F****igure S10. Performance evaluation of the sensor under simulated human environments.** Experimental setup and real-time electrical signals of the sensor during 23-min immersion in (a) artificial sweat and (b) acidic solution. (c) Bending response (0°–90°) of the sensor after exposure to acidic and saline environments.


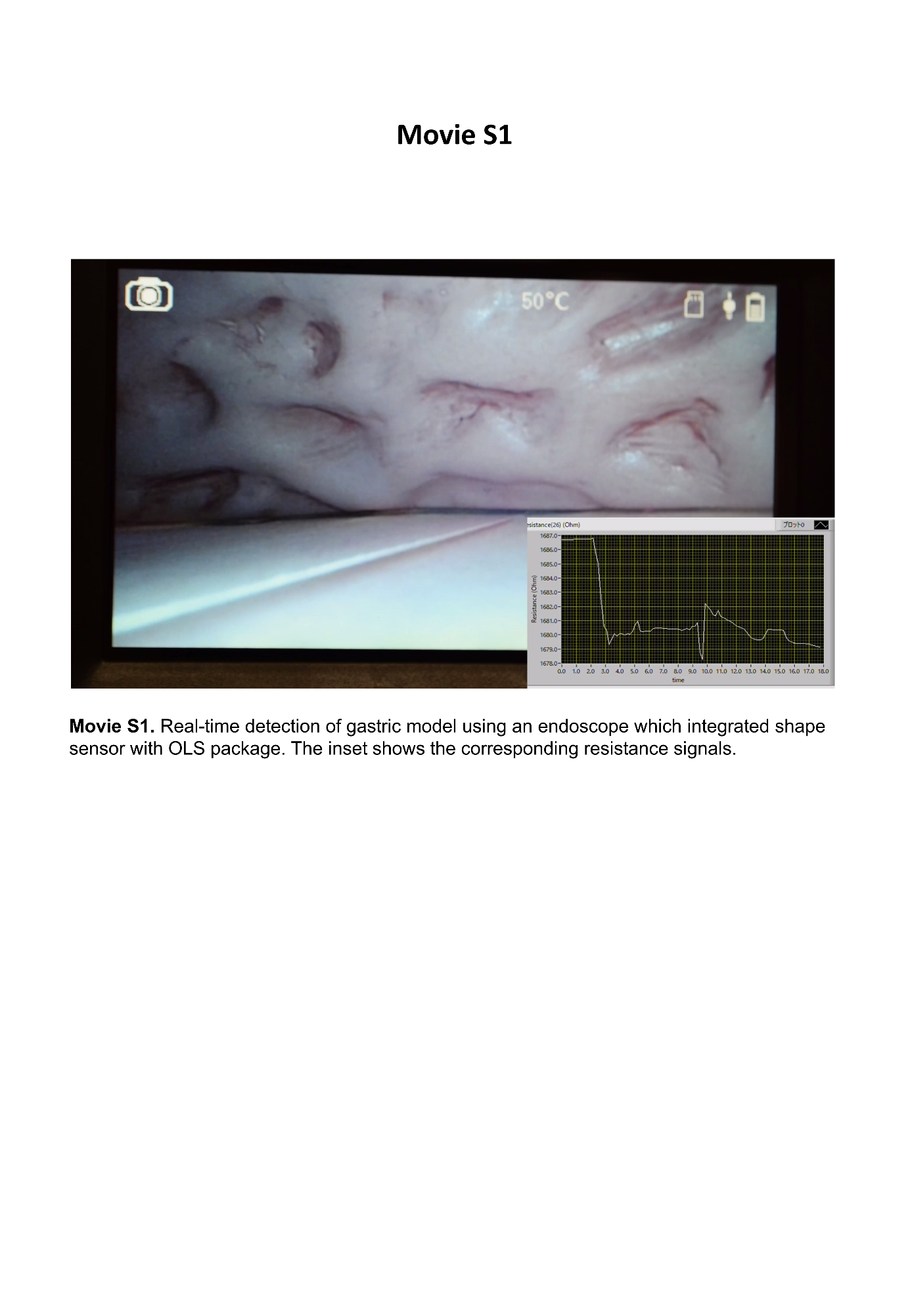


**Movie S1.** Real-time detection of a gastric model using an endoscope integrated with the OLS-packaged shape sensor. The inset shows the corresponding resistance signals.

**References**

[S1] S. Takamatsu, S. Goto, M. Yamamoto, T. Yamashita, T. Kobayashi, T. Itoh, *Sci. Rep.*, 2019, 9, 1893.

[S2] H. Liu, M. Takakuwa, M. Yamamoto, S. Nakashima, Z. Jiang, T. Yokota, T. Someya, T. Itoh, S. Takamatsu, *npj Flex. Electron.* 2025, in press.

[S3] S. P. Timoshenko, J. M. Gere, *Theory of Elastic Stability*, McGraw‐Hill, New York 1961.

[S4] R. M. Jones, *Mechanics of Composite Materials*, CRC Press, Boca Raton 2021.
